# DNA Methylation Analysis of the *Citrullus lanatus* Response to *Cucumber Green* *Mottle Mosaic Virus* Infection by Whole-Genome Bisulfite Sequencing

**DOI:** 10.3390/genes10050344

**Published:** 2019-05-07

**Authors:** Yuyan Sun, Min Fan, Yanjun He

**Affiliations:** Institute of Vegetables, Zhejiang Academy of Agricultural Sciences, Hangzhou 310021, China; syy1111@126.com (Y.S.); hyj1009@163.com (Y.H.)

**Keywords:** watermelon, CGMMV, WGBS, DNA methylation, RNA-seq

## Abstract

DNA methylation is an important epigenetic mark associated with plant immunity, but little is known about its roles in viral infection of watermelon. We carried out whole-genome bisulfite sequencing of watermelon leaves at 0 h (ck), 48 h, and 25 days post-inoculation with *Cucumber green mottle mosaic virus* (CGMMV). The number of differentially methylated regions (DMRs) increased during CGMMV infection and 2788 DMR-associated genes (DMGs) were screened out among three libraries. Most DMRs and DMGs were obtained under the CHH context. These DMGs were significantly enriched in the Kyoto Encyclopedia of Genes and Genomes (KEGG) pathways of secondary biosynthesis and metabolism, plant–pathogen interactions, Toll-like receptor signaling, and ABC transporters. Additionally, DMGs encoding PR1a, CaMs, calcium-binding protein, RIN4, BAK1, WRKYs, RBOHs, STKs, and RLPs/RLKs were involved in the watermelon–CGMMV interaction and signaling. The association between DNA methylation and gene expression was analyzed by RNA-seq and no clear relationship was detected. Moreover, downregulation of genes in the RdDM pathway suggested the reduced RdDM-directed CHH methylation plays an important role in antiviral defense in watermelon. Our findings provide genome-wide DNA methylation profiles of watermelon and will aid in revealing the molecular mechanism in response to CGMMV infection at the methylation level.

## 1. Introduction

*Cucumber green mottle mosaic virus* (CGMMV) is a member of the *Tobamovirus* genus that produces typical mosaic patterning on infected plants. It was first reported in England in 1935 [1]. Since then, the diversity of CGMMV strains, including CV3, CV4, watermelon strain, Yodo strain, Indian C strain, and muskmelon strain have been found to cause serious diseases in cucurbits, diminishing their fruit yields and quality [1,2,3,4,5]. The disease cycle of CGMMV is complex, involving two viral dispersion components which occur in the field or conservation tillage systems and within seedling nurseries [6]. Over the past five years, CGMMV has spread rapidly and is now reported in more than 30 countries and regions including Australia and North America [7,8,9,10,11]. In CGMMV-infected watermelon, mottling and mosaicism develop on the leaves of young plants, brown necrotic lesions occur in the stems and peduncles, and sponginess, rotting, and dirty-red discolorations are seen in the flesh [6]. Currently, CGMMV management primarily relies on measures of cultural, biological, and host resistance [6]. Therefore, understanding the associated host response mechanism will aid in the development of potential control methods for CGMMV management. 

Several studies have investigated interactions between cucurbits and CGMMV [12,13,14,15,16,17,18]. The transcriptome analysis of watermelon fruits and leaves identified genes that were differentially expressed in response to CGMMV infection, which were mainly involved in pathways of photosynthesis, plant–pathogen interactions, secondary metabolism, and plant hormone signal transduction [12,13]. Additionally, high-throughput microRNA (miRNA) sequencing analyses of CGMMV-infected cucumber and watermelon leaves revealed potential miRNAs that were activated in response to CGMMV infection [14,15]. Moreover, profiles of CGMMV-derived small interfering RNAs (siRNAs) in cucumber [16], bottle gourd [17], and watermelon [18] detected virus-derived siRNAs that were associated with interactions of CGMMV and cucurbits. 

DNA methylation is an important epigenetic mark involved in genome stability, transcriptional inactivation, and environmental responses [19,20]. In plants, DNA methylation occurs in the sequence contexts of CG, CHG, and CHH (H=A/T/C) [21]. Methylated CG (mCG) is catalyzed and maintained by methyltransferase 1 (MET1), while mCHG is catalyzed and maintained by chromomethylase 3 (CMT3) or CMT2 [22,23]. The maintenance of mCHH requires CMT2 and domains rearranged methyltransferases (DRM1 and DRM2) through the RNA-directed DNA methylation pathway, which require formation of double-stranded RNAs (dsRNAs) by two plant-specific homologs of RNA polymerase IV (Pol IV) and RNA-dependent RNA polymerase 2 (RDR2), then procession by Dicer-like 3 (DCL3) and Argonaute 4 (AGO4) or AGO6 to guide the DNA methylation [24]. The function of DNA methylation varies according to the genome location [25,26]. For example, DNA methylation of regulatory elements alters the chromatin structure, blocks transcriptional initiation, and downregulates gene expression. Conversely, methylation of repeat sequences is linked to silencing effects, and methylation of gene bodies is positively correlated with active transcription [25,26].

The dynamics of DNA methylation are involved in plant growth and development as well as in plant response to environmental stresses. Studies have suggested that DNA methylation regulates leaf morphology, flowering time, fertility, embryogenesis, and seed development in plants [27,28,29,30,31]. Moreover, DNA methylation is associated with plant response to environmental stresses. For instance, whole-genome bisulfite sequencing of single-cell root hairs and multicellular stripped roots in response to heat stress in soybean showed hypomethylation after the heat stress [32]. In *Arabidopsis*, differentially methylated regions (DMRs) were observed in drought-exposed lineages compared with control plants, suggesting that DNA methylation is relatively stable under drought stress [33]. 

DNA methylation is also dynamically involved in the mechanism of plant immunity [34,35]. In *Arabidopsis*, enhanced defense responses to *Pseudomonas syringae* pv. *tomato* (*Pst*) DC3000 were observed when immune-response genes involved in DNA methylation and demethylation were mutated [36,37]. In wheat, DMRs with CHH hypomethylation were revealed following *Blumeria graminis* f. sp. *tritici* (*Bgt*) infection. Argonaute 4 was also significantly downregulated, resulting in a substantial reduction in AGO4a-sorted 24-nt siRNA levels, especially for genes located near transposable elements (TEs) [35]. *Tomato yellow leaf curl virus* (TYLCV) V2 interacts with its host HDA6 and interferes with the recruitment of MET1 by HDA6, resulting in decreased methylation of the viral DNA genome by transcriptional gene silencing with a concomitant increase in host susceptibility to TYLCV infection [38]. In tomato, *Ty-1* confers resistance to geminiviruses by increasing cytosine methylation of the viral genomes, suggestive of enhanced transcriptional gene silencing [39]. 

RNA silencing (or RNA interference, RNAi) is a mechanism that induces messenger RNA (mRNA) degradation or inhibits translation at either the post-transcriptional level (PTGS) or the transcriptional level (TGS) which is involved in the RdDM pathway [40]. RNAi functions as an antiviral mechanism in plants and invertebrates [41]. Studies have shown that multiple DCLs, RDRs, and AGOs direct the antiviral RNAi defense in plants [39,41,42]. In addition, antiviral RNAi defense is suggested to mostly rely on a methylation-based defense, a process that involves the action of siRNA-directed methylation pathway component AGO4 [34,39,42].

Bisulfite sequencing has been used in analyzing DNA methylation in plant response to viral infection. For instance, reduced representation bisulfite sequencing (RRBS) of *cucumber mosaic virus* (CMV)-infected *Nicotiana tabacum* revealed that DMRs are enriched within CHH sequence contexts and are mainly located on the gene body [34]. Deep sequencing of bisulfite-treated DNA (BS-Seq) on TYLCV-infected tomato identify different epigenetic scenarios in the viral genome, suggesting that DNA methylation plays roles in plant defense and viral gene regulation [43]. Currently, whole-genome bisulfite sequencing (WGBS) has emerged as a powerful approach for studying DNA methylation by providing genome-wide methylation profiles at single-base resolution [44]. Whole-genome bisulfite sequencing has been successfully applied to profiling the DNA methylation in several plants, which reveals DNA methylation in controlling male sterility in *Brassica napus* [45], and regulating daily gene expression in *Populus nigra* and *Populus trichocarpa* [46,47]. However, WGBS application in studying DNA methylation dynamics in watermelon response to viral infection has not been reported. To investigate the profiles of DNA methylation and possible function of cytosine methylation in response to CGMMV infection in watermelon, we performed WGBS on watermelon leaves before and after CGMMV inoculation. Our findings identified DMRs and DMR-associated genes (DMGs) in response to CGMMV infection and reveal the possible role of DNA methylation in antiviral defense, which could be exploited to improve disease resistance in watermelon.

## 2. Plants and Materials

### 2.1. Plant Materials and Cucumber Green Mottle Mosaic Virus Inoculation

Seeds of the watermelon advanced inbred line “JJZ-M”, which is susceptible to CGMMV, were planted in a greenhouse and kept at 25 °C. Seedlings at the two-true leaf stage were inoculated with CGMMV. Leaves were separately harvested at 0 h (ck), 48 h post-inoculation (hpi), and 25 days post-inoculation (dpi). Leaves from three plants were mixed as a sample. Presence of CGMMV was verified by RT-PCR using specific primers of the CGMMV coat protein (F: 5’-ATGGCTTACAATCCGATCACAC-3’; R: 5’-CTAAGCTTTCGAGGTGGTAGCC-3’).

### 2.2. DNA Extraction

Genomic DNA samples were isolated from watermelon leaves using E.Z.N.A.^®^ Tissue DNA Kit (Omega Bio-tek, Norcross, GA, USA) according to the manufacturer’s instructions, and quality control was subsequently carried out on purified DNA samples. Genomic DNA was quantified using the TBS-380 fluorometer (Turner BioSystems Inc., Sunnyvale, CA, USA). High-quality DNA samples (OD 260/280 = 1.8–2.0, > 6 µg) were used to construct the fragment library.

### 2.3. Library Preparation and Illumina HiSeq Sequencing

Before bisulfite treatment, 25 ng lambda DNA was added to 5 µg genomic DNA from watermelon leaves. The mixed DNA was then fragmented to 450 bp with a sonicator (Sonics & Materials Inc., Danbury, CT, USA). After blunt ending and the 3′-end addition of dA, Illumina methylated adapters were added according to the manufacturer’s instructions by the Paired-End DNA Sample Prep Kit (Illumina, San Diego, CA, USA). The bisulfite conversion of genomic DNA was carried out using the ZYMO EZ DNA Methylation-Gold Kit (Zymo, Irvine, CA, USA), and amplified by 12 cycles of PCR using KAPA HiFi HotStart Uracil + ReadyMix (2×) (KAPA Biosystems, Boston, MA, USA). Ultra-high-throughput pair-end sequencing was carried out using Illumina HiSeq X Ten according to the manufacturer instructions at Biozeron Biotechnology Co., Ltd. (Shanghai, China) and 150-bp paired-end reads were generated. Raw HiSeq sequencing data were processed by the Illumina base-calling pipeline (SolexaPipeline-1.0).

Whole-genome bisulfite sequencing data from this study can be accessed at sequence read archive (SRA) database from NCBI (https://www.ncbi.nlm.nih.gov/sra) with accession codes No.: SRR8797403 (ck), SRR8797402 (48 h), and SRR8797401 (25 d).

### 2.4. Reads Quality Control and Mapping 

Raw paired-end reads were trimmed and quality controlled by Trimmomatic with default parameters. Clean BS-Seq reads were mapped to the reference genome of watermelon “Charleston Gray” with the Bisulfite Sequence Mapping Program (BSMAP) aligner allowing up to two mismatches to detect the methylation pattern of each cytosine in the genome [48]. The BSMAP uses the positions of all Cs in the reference sequences and applies bitwise masking to implement asymmetric C/T transition: T in bisulfite reads can be mapped to either C or T in the reference but not vice versa [48]. Alignments from both strands were combined, and for each read only the optimal alignments were kept. Multi-aligned reads, which were mapped to the watermelon genome at more than one location, were abandoned. Finally, the methylation status of each cytosine in watermelon genome was calculated on the basis of the alignments [49]. 

### 2.5. Identification of Methylated Cytosine Sites

The binomial test was performed for each cytosine base in the watermelon genome to check whether the cytosine site can be called a methylated cytosine site. Binomial probability values were then adjusted for multiple tests using false discovery rate (FDR). Cytosine sites with FDR < 0.01 were defined as methylated cytosine sites, as described previously [49].

### 2.6. Identification of Differentially Methylated Regions and DMR-Associated Genes

The methylation level of each cytosine was defined as the proportion of reads showing mC among all reads covering the same cytosine. The methylation level of a region was defined as the average methylation level of all Cs in this region. Only cytosines covered with at least four reads in a library were considered to identify DMRs. DMRs were identified using a 200-bp sliding window with 50 bp as a step-size. Cytosines (Cs) or thymines (Ts) were counted separately in each sliding window for three sequence contexts (CG, CHG, or CHH). The methylation level for a sliding window was determined as follows: methylation level = Σai/(Σai + bi), in which ai was the number of Cs and bi was the number of Ts mapping to the C sites [35]. DNA methylation levels of different libraries were compared pairwise using Fisher’s exact test, and p-values were adjusted for multiple comparisons using the Benjamini–Hochberg method. Windows with a FDR less than 0.05 and a change in methylation level more than 1.5-fold were retained for further analysis. The p-value of each cytosine in selected regions was calculated by Fisher’s exact test. 

Differentially methylated cytosines (DMCs) were identified if *p* ≤ 0.01 and FC ≥ 2 with absolute methylation differences of 0.4, 0.2, or 0.1 for CG, CHG, or CHH, respectively. Regions were only retained if they contained at least seven DMCs. Neighboring DMRs were combined if the gap was less than or equal to 100 bp [50]. Genes overlapping with significant DMRs by at least 1 bp in the functional region were defined as DMR-associated genes (DMGs) as described in a previous study [34]. In this study, seven functional regions, including intron, 2 kb upstream of genes (up-2k), 2 kb downstream of genes (down-2k), gene body, coding sequence (cds), 3ʹ untranslated region (utl3), and 5ʹ untranslated region (utl5), were divided based on gene structures of genome.

### 2.7. Kyoto Encyclopedia of Genes and Genomes Pathway Analysis of DMR-Associated Genes 

Differentially methylated regions associated genes were tested for Kyoto Encyclopedia of Genes and Genomes (KEGG) pathway enrichment using the hypergeometric test (Fisher’s exact test) with a 0.05 FDR correction. Pathways with *p* < 0.05 were considered significantly enriched [51].

### 2.8. Data Visualization

Genome-wide expression and DNA methylation profiles were visualized in Integrative Genomics Viewer (IGV) [52]. 

### 2.9. RNA Sequencing and Data Analysis

To analyze the relationship between DNA methylation and gene expression, watermelon leaves were also collected for RNA extraction at 0 h, 48 h, and 25 d post-CGMMV inoculation. Total RNA was extracted using TRIzol reagent (Invitrogen, Carlsbad, CA, USA) following the manufacturer’s procedure. Total RNA was subjected to Poly(A) mRNA isolation using poly-T oligo-attached magnetic beads (Invitrogen). The mRNA was fragmented into small pieces and then reverse-transcribed to create the final cDNA library. The paired-end sequencing was carried out on an Illumina HiSeq 4000 platform. After the transcriptome data was generated. StringTie was used to determine expression levels for mRNAs by calculating fragments per kilobase of exon per million reads (FPKM) [53]. 

## 3. Results

### 3.1. Phenotypic Observation of Watermelon Leaves Before and After Cucumber Green Mottle Mosaic Virus Infection

**Figure 1:**
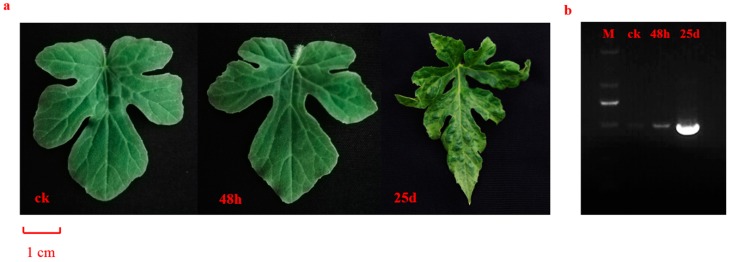
Phenotypes and verification of watermelon leaves before and post *Cucumber green mottle mosaic virus* (CGMMV) infection. (**a**) Phenotypes of watermelon leaves before and after CGMMV infection. Leaves from left to right were ck (control), 48 h, and 25 d post CGMMV infection, respectively. The scale bar is 1 cm for the watermelon leaves. (**b**) RT-PCR analysis of CGMMV presence for ck, 48 hpi (hours post-inoculation), and 25 dpi (days post-inoculation). Lanes from left to right were DL2000 marker, fragments of ck, 48 hpi, and 25 dpi, respectively.

At 25 dpi, characteristic symptoms of CGMMV, including mottling and mosaicism on the leaves and shriveling of veins, were observed in CGMMV-infected plants, while 48 h-infected plants showed no obvious disease symptoms compared with control (ck) plants (Figure 1a). We further verified the presence of CGMMV using the RT-PCR analysis. Specific 486-bp fragments were amplified in both 48 hpi and 25 dpi plants (Figure 1b), which suggest CGMMV begun to accumulate at 48 hpi and highly accumulated at 25 dpi. 

### 3.2. Profiles of Genome-Wide DNA Methylation in Watermelon Leaves

Leaves of ck, 48 hpi, and 25 dpi plants were collected for the construction of bisulfite-treated genomic DNA libraries. In total, WGBS generated 91,070,116, 80,314,902, and 73,989,862 raw reads by paired-end sequencing for ck, 48 hpi, and 25 dpi libraries, respectively, which obtained a total of 36,806,232,000 raw bases and 32,900,506,657 clean bases for three libraries. Of the 83,839,958 clean reads from the ck library, 90.41% (75,798,928) were uniquely mapped to the reference genome of watermelon “Charleston Gray” (http://cucurbitgenomics.org/organism/4), while of the 71,659,074 clean reads from the 48 hpi library and 67,011,742 clean reads from the 25 dpi library, 89.35% (64,029,094) and 90.13% (60,394,949) were uniquely mapped to the reference genome, respectively. Totals of 104,845, 160,432, and 150,563 clean reads with unknown bases (Ns) were obtained for the three libraries, respectively, and Q20 and Q30 percentages were approximately 97.0% and 90.0%, respectively, for all three libraries. Coverage of sequencing data were 82.05%, 74.54%, and 72.33% for ck, 48 hpi, and 25 dpi libraries. The CG percentages were 19.87%, 19.79%, and 20.29%, average read depths were 12.06, 10.49, and 11.62, and 60.73%, 51.57%, and 54.15% cytosines were covered by at least four reads in the watermelon genome, respectively, for ck, 48 hpi, and 25 dpi libraries. The depth and density of the sequencing were sufficient for high-quality genome-wide methylation analysis. Meanwhile, the bisulfite conversion efficiencies represented by the lambda DNA added into the libraries were 99.61%, 99.60%, and 99.64% for ck, 48 hpi, and 25 dpi libraries, respectively, indicating reliable results for the WGBS in the present study (Table 1). 

**Figure 2:**
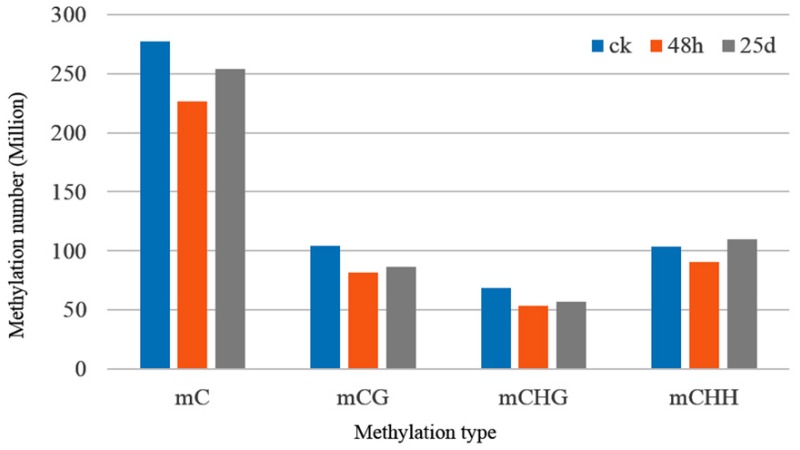
Numbers of mC, mCG, mCHG and mCHH identified in each sample by referring to the genome of watermelon “Charleston Gray”. “m” stands for methylated in the bases: C, G, H (either A, T or G)

**Figure 3:**
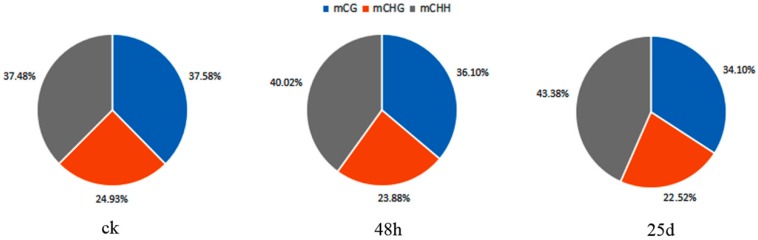
Percentages of methylated cytosines in each sample of watermelon leaves under each context.

**Figure 4:**
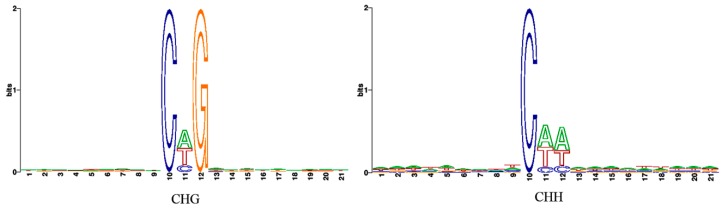
Sequence characteristic analysis of nine bp bases neighbouring methylated CHG and CHH. The *x*-axis represents the nine bp upstream and downstream of CHG and CHH, the height of the *y*-axis is the maximum entropy for the given sequence type (A, T, C, and G).

Genome-wide methylated cytosines totaled 276.95, 226.35, and 254.04 million for ck, 48 hpi, and 25 dpi libraries, respectively, by referring to the genome of watermelon “Charleston Gray” using BSMAP with a binomial test (Figure 2). Compared with ck, the number of methylated cytosines under CG, CHG, and CHH contexts decreased in the 48 hpi library, then increased in the 25 dpi library (Figure 2). Of note, CHG had the lowest methylated cytosines among all sequence contexts. In the ck library, the cytosine methylation percentage in the CHH context was similar to that in the CG context. However, the cytosine methylation percentage increased in the CHH context, while percentages of methylated cytosines in CG and CHG contexts decreased during CGMMV infection (Figure 3). In order to analyze the distribution of bases near methylated C of non-CG sites and calculate the probability of methylated types, sequence characteristics of 9 bp bases neighboring methylated CHG and CHH were analyzed using WebLogo (http://weblogo.berkeley.edu/logo.cgi). Bases neighboring methylated C of CHG were A(T/C)G and bases neighboring methylated C of CHH were A(T/C)A(T/C) in three libraries (Figure 4). 

**Figure 5:**
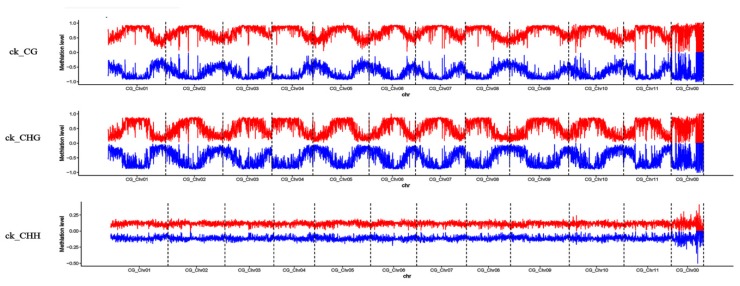

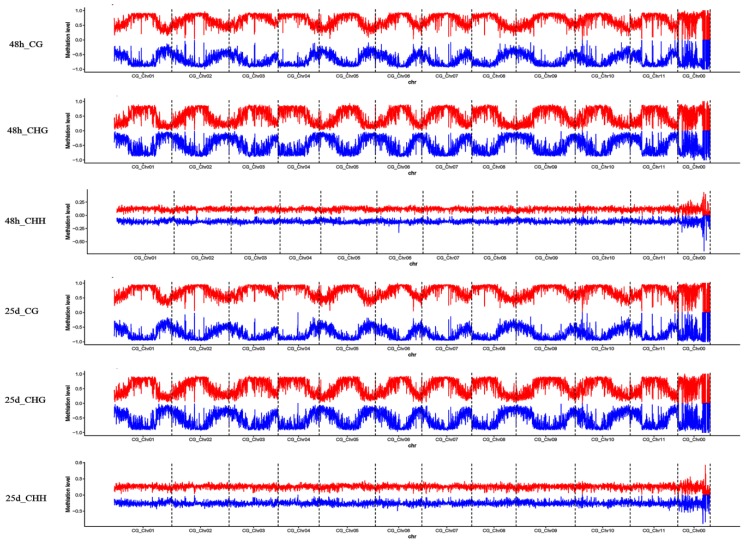
Chromosomal distribution of the global DNA methylation patterns in each sample under CG, CHG, and CHH contexts. CG_Chr00–CG_Chr11 in the *x*-axis represents chromosome 0–11 of the watermelon “Charleston Gray”. The *y*-axis represents the methylation level of DNA methylation patterns. The red lines indicate the methylation level in the sense strand of the watermelon genome and the blue lines indicate the methylation level in the antisense strand of the watermelon genome.

**Figure 6:**
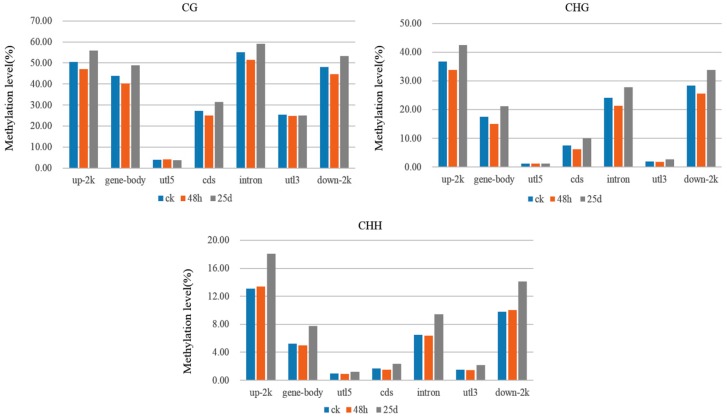
DNA methylation levels in different genomic functional regions of the watermelon genome. The *x*-axis represents the genomic regions in the watermelon genome. utl5: 5ʹ untranslated region, cds: coding sequence, utl3: 3ʹ untranslated region, up-2k: 2 kb upstream of genes, down-2k: 2 kb downstream of genes. The *y*-axis represents the methylation levels for ck, 48 hpi, and 25 dpi libraries under CG, CHG, and CHH contexts.

To present the global DNA methylation profiles of the three libraries, we further analyzed methylation levels and found them to be unevenly distributed throughout the watermelon chromosomes under CG, CHG, and CHH contexts (Figure 5). Dividing the genome into seven functional regions based on gene structures, including the gene body, 5ʹ untranslated region (utl5), coding sequence (cds), intron, 3ʹ untranslated region (utl3), 2 k upstream (up-2k), and 2 k downstream (down-2k) of genes, revealed the highest methylation level for up-2k and lowest for utl5 under CHG and CHH contexts (Figure 6). However, under the CG context, the highest methylation was observed in intron, followed by up-2k, down-2k, gene body, cds, utl3, and utl5 (Figure 6). 

### 3.3. Detection of Differentially Methylated Regions

**Figure 7:**
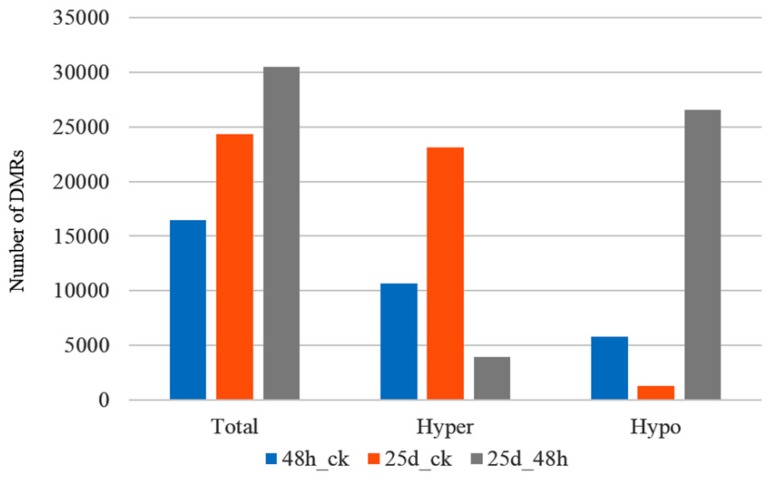
Number of differentially methylated regions (DMRs) identified in 48h_ck, 25d_ck, and 25d_48h.

Typically, the number of DMRs increased during CGMMV infection in watermelon leaves (Figure 7). Additionally, the number of hyper DMRs was highest in 25d_ck and lowest in 25d_48h, while hypo-DMRs were highest in 25d_48h and lowest in 25d_ck (Figure 7). We observed a total of 16,483 DMRs (10,683 hypermethylated and 5800 hypomethylated) for 48h_ck, 24,350 (23,086 hypermethylated and 1264 hypomethylated) for 25d_ck, and 30,523 (3949 hypermethylated and 26,574 hypomethylated) for 25d_48h (Table 2). Of these, 205, 205, and 16,073 were identified for 48h_ck, 61, 96, and 24,193 for 25d_ck, and 181, 215, and 30,127 for 25d_48h under CG, CHG, and CHH contexts, respectively (Table 2 and Table S1). Most DMRs were located in intergenic regions of the watermelon genome (Table 2 and Table S1). The fact that more DMRs were detected under the CHH context than under CG and CHG contexts implies that DNA methylation of CHH plays an important role in the CGMMV response. 

### 3.4. Detection of DMR-Associated Genes

**Figure 8:**
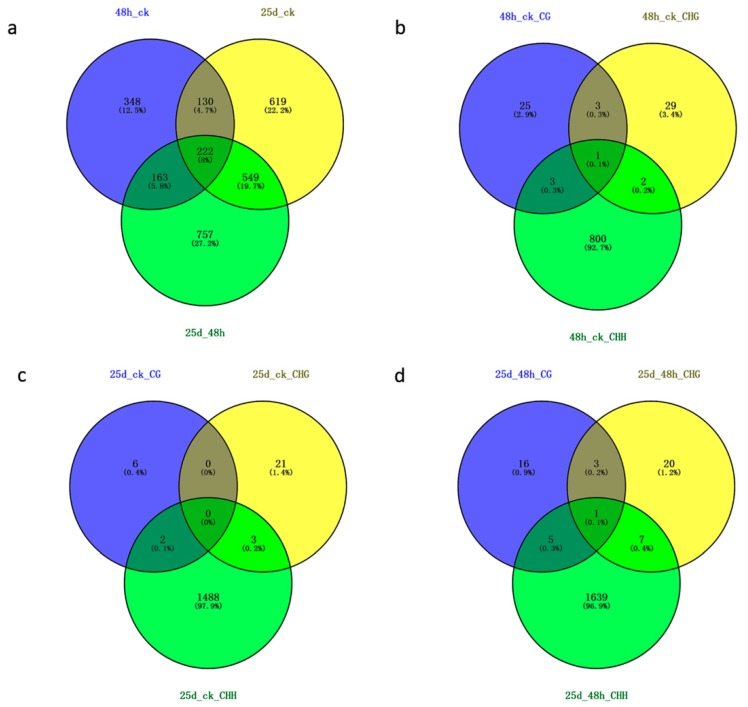
Venn diagram of DMR-associated genes (DMGs) among three libraries. (**a**) Venn diagram of DMGs among 48h_ck, 25d_ck, and 25d_48h; (**b**) Venn diagram of DMGs for 48h_ck under CG, CHG, and CHH contexts; (**c**) Venn diagram of DMGs for 25d_ck under CG, CHG, and CHH contexts; (**d**) Venn diagram of DMGs for 25d_48h under CG, CHG, and CHH contexts.

A total of 2788 DMGs were screened out among three libraries, including 863 for 48h_ck, 1520 for 25d_ck, and 1691 for 25d_48h (Figure 8a). A total of 222 DMGs were common to all three libraries, 130 were common to 48h_ck and 25d_ck, 163 were common to 48h_ck and 25d_48h, and 549 were common to 25d_ck and 25d_48h (Figure 8a). Additionally, 32 (seven hyper and 25 hypo), 35 (10 hyper and 25 hypo), and 806 (561 hyper and 245 hypo) DMGs were identified for 48h_ck, eight (two hyper and six hypo), 24 (18 hyper and six hypo), and 1493 (1475 hyper and 18 hypo) DMGs were identified for 25d_ck, and 25 (four hyper and 21 hypo), 31 (31 hypo), and 1652 (58 hyper and 1594 hypo) DMGs were identified for 25d_48h under the contexts of CG, CHG, and CHH, respectively (Figure 8b–d; Table S2). Consistent with DMR findings, many more DMGs were detected under the CHH context than under CG and CHG contexts. 

### 3.5. Functional Analysis of DMR-Associated Genes

**Figure 9:**
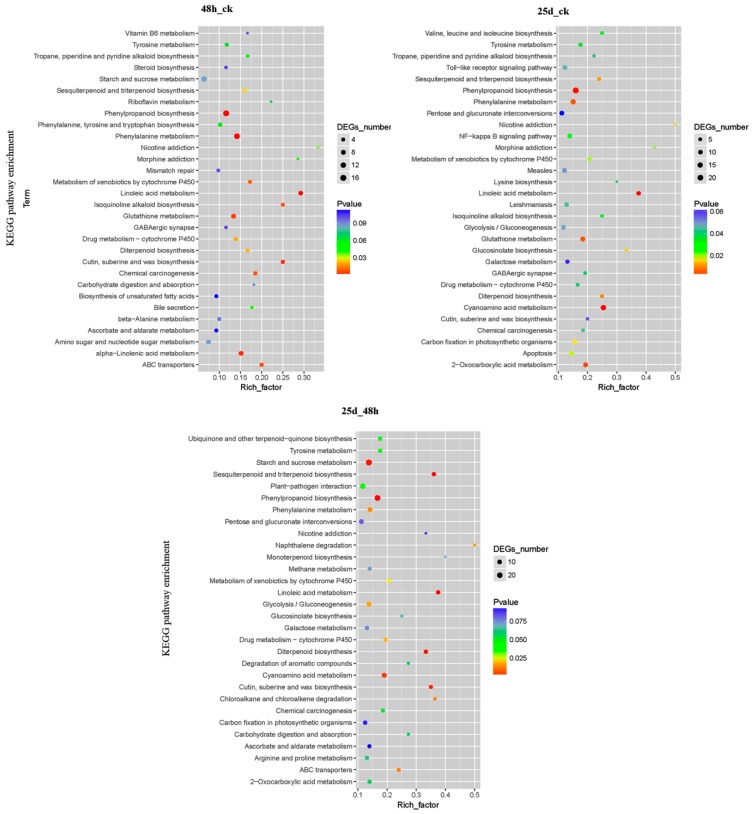
Statistics of the top 20 pathways enriched for DMGs in 48h_ck, 25d_ck, and 25d_48h. The size of each circle represents the number of DMGs enriched in the corresponding pathway. The enrichment factor was calculated using the number of enriched genes divided by the total number of background genes in the corresponding pathway. The *p*-value was calculated using the Benjamini–Hochberg correction. A pathway with *p* < 0.05 was considered significantly enriched.

A total of 196, 234, and 232 KEGG pathways for DMGs were identified in 48h_ck, 25d_ck, and 25d_48h libraries, respectively (Table S3). Among these, 14, 23, and 21 KEGG pathways with *p* < 0.05 were significantly enriched, respectively (Figure 9 and Table S4). For example, phenylalanine metabolism (ko00360), phenylpropanoid biosynthesis (ko00940), cutin, suberine, and wax biosynthesis (ko00073), linoleic acid metabolism (ko00591), the Toll-like receptor signaling pathway (ko04620), starch and sucrose metabolism (ko00500), plant–pathogen interactions (ko04626), and ABC transporters (ko02010) were significantly enriched during CGMMV infection (Figure 9 and Table S3).

### 3.6. DMR-Associated Genes Related to the Cucumber Green Mottle Mosaic Virus Response in Watermelon Leaves

In this study, a few DMGs were invovled in the KEGG pathways of secondary biosynthesis and metabolism, plant–pathogen interactions, Toll-like receptor signaling, and ABC transporters. Among them, 15 DMGs, encoding proteins of sucrose synthase, 4-coumarate--CoA ligase, linoleate 9S-lipoxygenase, pectinesterase, WRKY, calmodulin-like protein, pathogenesis-related protein, RPM1-interacting protein, brassinosteroid insensitive 1-associated receptor kinase, and ABC transporter were selected for visualization the dynamics of genic DNA methylation using the IGV (Figure S1).

#### 3.6.1. DMR-Associated Genes Involved in Secondary Biosynthesis and Metabolism

A subset of DMGs were involved in the pathways of secondary biosynthesis and metabolism, including phenylpropanoid biosynthesis and metabolism (ko00360; ko00940), cutin, suberine and wax biosynthesis (ko00073), linoleic acid metabolism (ko00591), and starch and sucrose metabolism (ko00500) (Table S4). Most of these DMGs were hypermethylated for 25d_ck and hypomethylated for 25d_48h (Table S4).

A total of 43 DMGs were involved in phenylpropanoid biosynthesis (ko00940) and phenylpropanoid metabolism (ko00360) pathways (Table S4). Of these, four DMGs (*ClCG09G003320*, *ClCG10G012970*, *ClCG10G012990*, and *ClCG10G013000*) encoding cinnamoyl-CoA reductase (CCR) were identified. Both *ClCG09G003320* (CCR1) and *ClCG10G012970* (CCR2) were hypermethylated for 25d_ck and hypomethylated for 25d_48h under the CHH context. *ClCG10G012990* (CCR2) was hypomethylated for 48h_ck under the CHH context. Another DMG (*ClCG10G013000*) encoding CCR2 was hypermethylated for 48h_ck and hypomethylated for 25d_48h under the CHH context. Additionally, two DMGs (*ClCG07G002280* and *ClCG11G012560*) encoded 4-coumarate-CoA ligase. *ClCG07G002280* was hypermethylated for 25d_ck and hypomethylated for 25d_48h under the CHH context, and *ClCG11G012560* was hypomethylated for 25d_48h under the CHH context. Moreover, 17 DMGs encoding peroxidase were identified in this pathway, of which most were hypermethylated or hypomethylated under the CHH context. 

Eight DMGs (*ClCG01G012910*, *ClCG02G022090*, *ClCG03G000870*, *ClCG04G001450*, *ClCG05G002780*, *ClCG05G002790*, *ClCG06G000770*, and *ClCG11G009050*) were associated with cutin, suberine, and wax biosynthesis pathways (Table S4). These DMGs encoded fatty acyl-CoA reductase (FAR), cytochrome P450 (CYP), protein ECERIFERUM 1-like (CER1-like) and omega-hydroxypalmitate O-feruloyl transferase. *ClCG03G000870* (CYP), *ClCG11G009050* (omega-hydroxypalmitate O-feruloyl transferase), and *ClCG06G000770* (CER1-like) were hypomethylated for 25d_48h under the CHH context, *ClCG04G001450* (FAR2) was hypomethylated for 48h_ck under the CHH context, and *ClCG05G002780* (CER1-like) was hypermethylated for 25d_ck and hypomethylated for 25d_48h under the CHH context. Two *FAR3* genes (*ClCG01G012910* and *ClCG02G022090*) were commonly hypermethylated for 48h_ck and 25d_ck, and hypomethylated for 25d_48h under the CHH context.

Among the 13 DMGs involved in the linoleic acid metabolism pathway, 11 DMGs (*ClCG02G015640*, *ClCG02G015670*, *ClCG02G015680*, *ClCG02G015690*, *ClCG02G015700*, *ClCG02G015710*, *ClCG02G015730*, *ClCG02G015750*, *ClCG02G023560*, *ClCG02G023610*, and *ClCG09G007920*) encoded lipoxygenases (LOXs) (Table S4). In all cases under the CHH context, *ClCG02G015640* (linoleate 9S-LOX6-like) was hypermethylated for 25d_ck, and *ClCG02G023610* (LOX7) was hypermethylated for 25d_48h. *ClCG02G023560* (linoleate 13S-LOX2-1) and *ClCG09G007920* (linoleate 9S-LOX-like) were hypermethylated for 25d_ck, *ClCG02G015690* (linoleate 9S-LOX5) was hypermethylated for 48h_ck and 25d_ck, and *ClCG02G015750* (linoleate 9S-LOX5 isoform X2) was hypermethylated for 48h_ck and 25d_48h. *ClCG02G015680* and *ClCG02G015700* encoding linoleate 9S-LOX6-like were hypermethylated for 25d_ck and hypomethylated for 25d_48h, *ClCG02G015670* (LOX) was hypomethylated for 48h_ck and 25d_48h, *ClCG02G015710* (linoleate 9S-LOX6-like) was hypermethylated for 25d_ck and hypomethylated for 48h_ck and 25d_48h, and *ClCG02G015730* (linoleate 9S-LOX6-like isoform X1) was hypermethylated for 48h_ck and 25d_ck and hypomethylated for 25d_48h.

Forty DMGs were involved in the starch and sucrose metabolism pathway (ko00500) (Table S4). These mainly encoded pectinesterase (PE), hexokinase (HK), sucrose synthase (SUS), β-amylase, cellulose synthase (CesA), and β-glucosidase. Six DMGs (*ClCG03G014780*, *ClCG05G007400*, *ClCG06G007740*, *ClCG07G008840*, *ClCG09G005530*, and *ClCG09G020720*) encoded PEs, of which *ClCG09G020720*, *ClCG03G014780* and *ClCG07G008840* were hypermethylated for 48h_ck, 25d_ck, and 25d_48h under the CHH context. In all cases under the CHH context, *ClCG05G007400* and *ClCG09G005530* were hypomethylated for 48h_ck and 25d_48h, *ClCG06G007740* encoding PE29 was hypermethylated for 25d_ck and hypomethylated for 25d_48h, and *ClCG01G008480* encoding SUS5 was hypermethylated for 48h_ck and hypomethylated for 25d_48h. DMGs encoding β-amylase (*ClCG07G011170*) and CesA (*ClCG10G01117*0) were commonly hypermethylated for 25d_ck and hypomethylated for 25d_48h, and DMGs encoding HK1 (*ClCG11G002110*) and HK2 (*ClCG05G018300*) were hypomethylated for 25d_48h and hypermethylated for 48h_ck.

#### 3.6.2. DMR-Associated Genes Involved in Plant–Pathogen Interactions

Twenty-two DMGs were identified as participating in plant–pathogen interactions (ko04626) (Table S4). These mainly encoded pathogenesis-related protein (PR), respiratory burst oxidase homolog protein (RBOH), cyclic nucleotide-gated ion channel protein (CNGC), RPM1-interacting protein 4 (RIN4), BRASSINOSTEROID INSENSITIVE 1-associated receptor kinase 1 (BAK1), calmodulin (CaM)-like protein, calcium-binding protein, and WRKY transcription factors (TFs). Two DMGs (*ClCG07G007870* and *ClCG02G019200*) encoding WRKY22 and WRKY26 were hypomethylated for 48h_ck and 25d_ck under the CHH context, while DMG (*ClCG02G002690*) encoding WRKY1 was hypermethylated for 25d_ck and hypomethylated for 25d_48h under the CHH context. *ClCG02G005880* and *ClCG02G016580* encoding CaM-like 1 and CaM-like 8 were hypomethylated for 25d_48h under CG and CHH contexts, calcium-binding protein CML22 (*ClCG05G003310*) was hypomethylated for 25d_48h and calcium-binding protein CML31 (*ClCG03G008520*) was hypomethylated for 48h_ck and 25d_48h under the CHH context. Three *RBOHs* (*ClCG01G002290*, *ClCG03G017090*, and *ClCG07G007770*) and a *PR1a* gene (*ClCG02G00723*0) were hypomethylated for 25d_48h under the CHH context, while a *BAK1* gene (*ClCG09G015110*) was hypermethylated for 48h_ck and an *RIN4* gene (*ClCG05G003210*) was hypermethylated for 25d_ck and hypomethylated for 25d_48h under the CHH context.

#### 3.6.3. DMR-Associated Genes Involved in the Toll-like Receptor Signaling Pathway

Fourteen DMGs were involved in the Toll-like receptor signaling pathway (ko04620) (Table S4). Four of these (*ClCG02G020720*, *ClCG07G002710*, *ClCG09G010710*, and *ClCG11G011240*) encoded serine/threonine-protein kinases (STKs). *ClCG02G020720* was hypermethylated for 25d_ck under the CHH context and *ClCG07G002710* was hypomethylated for 48h_ck under the CG context. *ClCG09G010710* and *ClCG11G011240* were hypermethylated for 25d_ck and hypomethylated for 25d_48h under the CHH context. Six DMGs (*ClCG05G016550*, *ClCG05G017520*, *ClCG05G025640*, *ClCG07G014730*, *ClCG08G006270*, and *ClCG08G012560*) encoding receptor-like protein/kinases (RLPs/RLKs) were identified in this pathway. *ClCG05G016550*, *ClCG05G017520*, *ClCG07G014730*, and *ClCG08G012560* were commonly hypermethylated for 25d_ck and hypomethylated for 25d_48h under the CHH context, *ClCG08G006270* was hypomethylated for 25d_48h under the CHH context, and *ClCG05G025640* was hypomethylated for 48h_ck under the CG context, hypermethylated for 25d_ck under the CHH context, and hypomethylated for 25d_48h under CG and CHH contexts. 

#### 3.6.4. DMR-Associated Genes Involved in ABC Transporters

Six DMGs were involved in the ATP-Binding Cassette (ABC) transporter pathway (ko02010) (Table S4). *ClCG01G005380* encoding ABC transporter B family member 28 was hypermethylated for 25d_ck and hypomethylated for 25d_48h under the CHH context. One ABC transporter B family member 4 gene, *ClCG11G013200*, was hypermethylated for 48h_ck and hypomethylated for 25d_48h under the CHH context. Two DMGs (*ClCG02G018950* and *ClCG09G018580*) encoding ABC transporter B family members 15 and 25 were hypermethylated for 48h_ck under the CHH context. Another two DMGs (*ClCG02G007990* and *ClCG10G012900*), encoding ABC transporter B family members 13 and 19, were hypermethylated for 25d_ck and hypomethylated for 48h_ck and 25d_48h under the CHH context. 

### 3.7. Association between DNA Methylation and Gene Expression

**Figure 10:**
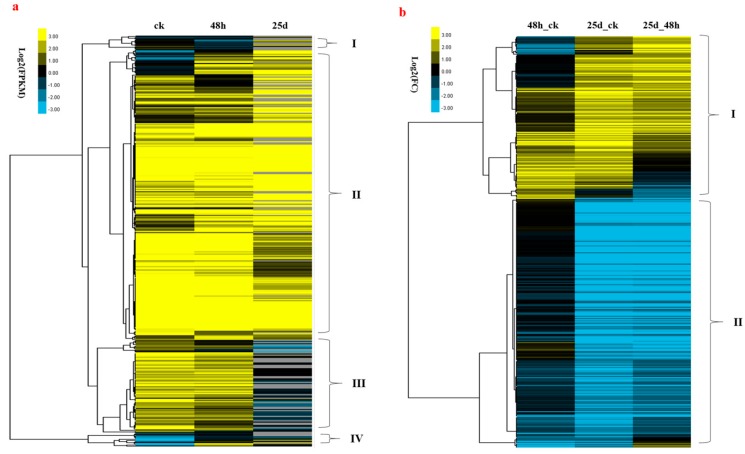
Hierarchical clustering of expression profiles for 362 differentially expressed DMGs among three libraries. (**a**) The log_2_(Fragments Per Kilobase of exon per Million reads) for ck, 48 hpi, and 25 dpi libraries; (**b**) the log_2_(FoldChange) for 48h_ck, 25d_ck, and 25d_48h. The trees were generated using the Cluster 3.0 (https://www.encodeproject.org/software/cluster/) and Java Treeview (http://jtreeview.sourceforge.net/).

Changes in methylation levels in genomic regions tend to be associated with alteration in gene expression [34]. To analyze the expression changes of DMGs, we carried out the RNA-sequencing (RNA-seq) analysis for watermelon leaves sampled at the same stages with WGBS. Among the 2788 DMGs, 1787 were detected and expressed (Table S5), and 362 of them showed differential expression among three libraries (Table S5 and Figure 10). These 362 DMGs were clustered into four clusters (I–IV) based on the log_2_(FPKM) for ck, 48 hpi, and 25 dpi libraries (Figure 10a). In addition, these 362 differentially expressed DMGs were mainly divided into two clusters according to the log_2_(FC) for 48h_ck, 25d_ck, and 25d_48h (Figure 10b). DMGs of Cluster І showed upregulated profiles, whereas DMGs of Cluster ІІ showed downregulated profiles during the process of CGMMV infection (Figure 10b).

Ten of the 38 (26.31%) DMGs under the CG context, 12 of the 47 (25.53%) DMGs under the CHG context, and 343 of the 1720 (19.94%) DMGs under the CHH context showed differential expression among three libraries (Table S5). The similar percentages of differentially expressed DMGs under CG, CHG, and CHH contexts indicated the expression changes did not correlate with the methylated sequence contexts. In detail, ten DMGs under the CG context, including *ClCG01G00163*0 (dof zinc finger protein DOF4.6-like), *ClCG01G003020* (protein P21-like), *ClCG02G001570* (scarecrow-like protein 32), *ClCG02G005880* (calmodulin-like protein 1), *ClCG02G018820* (peroxidase 7-like), *ClCG05G005770* (uncharacterized), *ClCG05G006560* (psbB), *ClCG10G001720* (transcriptional regulator SUPERMAN), *ClCG10G013330* (photosynthetic NDH subunit of subcomplex B2), and *ClCG11G013230* (uncharacterized protein) were differentially expressed during the CGMMV infection. Under the CHG context, twelve DMGs, such as *ClCG01G006770* (purple acid phosphatase 17), *ClCG01G006860* (hypothetical protein), *ClCG01G013770* (unannotated), *ClCG02G00190*0 (hypothetical protein), *ClCG03G007120* (coatomer subunit zeta-1-like), *ClCG04G002160* (anaphase-promoting complex subunit 6), *ClCG04G009510* (type-1 glutamine synthetase 1-like), *ClCG05G026970* (uncharacterized protein), *ClCG07G01716*0 (glycine-rich cell wall structural protein 2), *ClCG08G009690* (NADH dehydrogenase subunit 7), *ClCG09G013770* (hypothetical protein), *ClCG10G003690* (germacrene D synthase-like), showed differential expression. Under the CHH context, DMGs encoding cytochrome P450, chlorophyll a–b binding protein, leucine-rich repeats (LRR) receptor-like serine/threonine-protein kinase, cellulose synthase, respiratory burst oxidase homolog protein, calcium-binding protein, pectinesterase, ABC transporter, auxin transporter, NAC, and MYB transcription factor, were differentially expressed during the process of CGMMV infection. 

Moreover, the relationship between methylation type and gene expression was analyzed. For example, the hyper-methylated *ClCG01G001630* was downregulated for 48h_ck under CG context; however, under the same context, the hyper-methylated *ClCG01G003020* was upregulated for 48h_ck. Under the CHG context, *ClCG01G013770*, *ClCG02G001900*, and *ClCG03G007120* were hypo-methylated and the expression was commonly increased for 25d_48h, while the hypo-methylated *ClCG01G006770*, *ClCG04G009510*, and *ClCG05G026970* were decreased for 25d_48h. The results indicated that the hyper- or hypo-methylated genes did not have the correlation with gene upregulation or downregulation. 

### 3.8. Expression of Genes Invovled in the RdDM Pathway and RNA Interference in Watermelon

RdDM is the major small RNA-mediated epigenetic pathway in plants [24]. The dynamic expression of genes in the RdDM pathway, including *CMT*, *DRM2*, *RDR2*, *DCL3*, *AGO4,* and *AGO6*, were analyzed in present study (Table 3). Three *CMT* genes (*ClCG03G013970*, *ClCG10G001140* and *ClCG11G000400*) were detected and *ClCG03G013970* was significantly down-regulated at 48h_ck and up-regulated at 25d_48h. Two genes (*ClCG10G014050* and *ClCG06G004300*) encoding DRM2 expressed in three libraries. *ClCG06G004300* showed significant upregulation at 25 dpi compared with ck and 48 hpi, and *ClCG06G004300* gradually reduced the expression during the process of CGMMV infection. *ClCG06G016860* encoding RDR2 showed decreased expression during the process of CGMMV infection. Three genes (*ClCG08G009740*, *ClCG10G005250*, and *ClCG02G002110*) encoding DCL3 were detected. Among which, *ClCG08G009740* was slightly downregulated during the process of CGMMV infection, and *ClCG10G005250* and *ClCG02G002110* were slightly upregulated at 48 hpi, then downregulated at 25 dpi compared with ck. Moreover, *AGO4* (*ClCG02G024220*) and *AGO6* (*ClCG09G011170*) was significantly downregulated at 25 dpi compared with ck. Generally, genes involved in the RdDM pathway showed downregulation after the CGMMV infection, especially at the 25 dpi stage.

In addition, we also analyzed the expression of other genes encoding DCLs, RDRs, and AGOs that direct the antiviral RNAi defense in watermelon. *DCL2* (*ClCG03G010530*) was significantly upregulated at 25 dpi compared with ck and 48 hpi. Expression of *DCL4* (*ClCG06G012100*) slightly increased at 48 hpi, then decreased at 25 dpi compared with ck. Five genes (*ClCG00G004530*, *ClCG04G012520*, *ClCG05G023990*, *ClCG05G004800* and *ClCG01G014010*) encoding AGO proteins were detected and expressed. *ClCG00G004530* (AGO10) was gradually downregulated and *ClCG01G014010* (AGO) was gradually upregulated in the process of CGMMV infection. *ClCG04G012520* (AGO1B), *ClCG05G023990* (AGO), and *ClCG05G004800* (AGO) commonly decreased at 48 hpi, and then increased at 25 dpi. Four genes (*ClCG01G006600*, *ClCG01G006450*, *ClCG08G013320* and *ClCG07G005340*) encoding RDRs were expressed. Among them, *ClCG08G013320* (RDR6) significantly reduced the expression after infection with CGMMV.

## 4. Discussion

DNA methylation is a common feature of eukaryotic epigenomes. Many studies have improved our understanding of the variation in DNA methylation associated with the defense response to viral infection in plants, including *beet severe curly top virus* in *Arabidopsis* [54], TYLCV in tomato [39], and CMV in tobacco [34]. In this study, we tested the effect of global DNA methylation on CGMMV invasion in watermelon for the first time and determined its role during CGMMV infection. 

### 4.1. Global DNA Methylation Level and Genomic Distribution of DNA Methylation under Cucumber Green Mottle Mosaic Virus Infection

Global DNA methylation levels varied with different stages of the CGMMV infection, being highest in the ck library and lowest in the 48 h library (Figure 2). Additionally, the methylation density was highest under the CHH context, followed by CHG and CG contexts (Figure 2). This is consistent with a previous finding that methylated regions were enriched with CHH sequence contexts in response to CMV infection in tobacco [34]. In the present study, methylation levels were highest for the up-2k genomic region under CHG and CHH contexts (Figure 6). DNA methylation of promoters (up-2k) would lead to changes in gene expression by altering the chromatin structure and blocking transcriptional initiation [26]. However, under the CG context, the highest methylation level was observed within introns, followed by up-2k, down-2k, gene body, cds, utl3, and utl5 in all three libraries (Figure 6). DNA methylation of gene bodies is positively correlated with transcriptional activation and plays an important role in silencing repetitive elements and alternative splicing [25,26]. 

### 4.2. DMRs and DMGs Mainly Gathered under the CHH Context During Cucumber Green Mottle Mosaic Virus Infection 

The number of DMRs increased from 16,483 to 30,523 during the CGMMV infection, and hyper DMRs were highest in 25d_ck and lowest in 25d_48h, while hypo DMRs were highest in 25d_48h and lowest in 25d_ck (Figure 7). A total of 2788 DMGs were screened out of the three libraries, with 863, 1520, and 1691 detected for 48h_ck, 25d_ck, and 25d_48h, respectively (Table S2). Of note, the vast majority of DMRs and DMGs occurred under the CHH context, implying that DNA methylation in this state plays an important role in the response to CGMMV infection in watermelon. 

### 4.3. DMR-Associated Genes Enriched in Plant–Pathogen Interactions During Cucumber Green Mottle Mosaic Virus Infection

Within the plant–pathogen interaction, plants have evolved innate immune systems that recognize the presence of pathogens and initiate effective defense responses, whereas pathogens have evolved effector proteins that suppress host immune responses [55]. We found that DMGs encoding CaM-like protein, calcium-binding protein, WRKYs, PR1a, RBOHs, CNGCs, RIN4, and BAK1 were involved in plant–pathogen interaction pathways, and most of these genes were hypermethylated for 25d_ck and hypomethylated for 25d_48h (Table S4).

The Ca^2+^ signaling pathway plays an important role in plant defense to biotic and abiotic stresses [56]. In the present study, two DMGs (*ClCG03G008520* and *ClCG05G003310*) encoding calcium-binding proteins CML22 and CML31 were hypomethylated under the CHH context during the CGMMV infection, while DMGs *ClCG02G005880* and *ClCG02G016580*, encoding CaM-like protein 1 and CaM-like protein 8, were hypomethylated under CG and CHH contexts, respectively. CaM is the primary cellular Ca^2+^ receptor which recognizes and binds specific Ca^2+^ signals through the EF-hand domain and interacts with the calmodulin-binding domains of target calmodulin-binding proteins to transfer Ca^2+^ signals [57]. Many WRKY TFs have been demonstrated to associate with various defense responses in plants [58,59,60]. For example, *OsWRKY67* induces the transcription of a range of defense-related genes, including those involved in the salicylic acid-dependent pathway [58]. Moreover, *OsWRKY62* negatively regulates the basal and Xa21-mediated resistance to bacterial blight [59], and *OsWRKY28* is negatively correlated with blast disease resistance [60]. In this study, three DMGs (*ClCG02G002690*, *ClCG02G019200*, and *ClCG07G007870*) encoding WRKYs were identified. *ClCG02G002690* (WRKY1) was hypermethylated for 25d_ck and hypomethylated for 25d_48h under the CHH context, while *ClCG02G019200* (WRKY26) was hypermethylated for 25d_ck and *ClCG07G007870* (WRKY22) was hypomethylated for 48h_ck under the CHH context. These WRKYs might regulate the watermelon response to CGMMV infection.

The PRs are associated with the development of systemic acquired resistance against further infection enforced by fungi, bacteria, and viruses [61]. PR1a was the first PR-1 member to be purified and characterized [62], and *PR1a*-overexpressing plants exhibited increased tolerance to the oomycete pathogens *Phytophthora parasitica* var. nicotianae and *Peronospora tabacina* [63]. In this study, *ClCG02G007230* encoding PR1a was hypomethylated for 25d_48h under the CHH context, suggesting *ClCG02G007230* is associated with the CGMMV response in watermelon.

The RBOHs have been established as important second messengers that regulate the expression of hundreds of genes in response to stresses [64,65]. Three DMGs (*ClCG01G002290*, *ClCG03G017090*, and *ClCG07G007770*) encoding RBOHs were identified in this pathway in the present study, and all were hypomethylated for 25d_48h under the CHH context. *Arabidopsis* CNGCs were previously shown to be involved in plant defense responses against diseases, with *AtCNGC2* and *AtCNGC4* mutants (dnd1 and dnd2) demonstrating similar phenotypes such as impaired hypersensitive reactions and enhanced broad-spectrum resistance to bacterial pathogens [66,67]. Five DMGs (*ClCG04G009060*, *ClCG05G008780*, *ClCG05G008800*, *ClCG06G016900*, and *ClCG10G014650*) encoding CNGCs were detected in this pathway in the present study, and all were methylated under the CHH context. 

RIN4 is the only known protein that regulates both branches of the plant immune system. After pathogen-associated molecular pattern (PAMP) treatment, *RIN4* overexpression lines exhibited decreased callose deposition and enhanced growth of virulent and type III secretion-deficient *Pst*, indicating a reduction in pattern-triggered immunity [68]. Moreover, RIN4 associates with the C-terminal autoinhibitory domain of PM H^+^-ATPase to regulate the response of leaf stomata to PAMPs [69]. A *RIN4* gene (*ClCG05G003210*) was hypermethylated for 25d_ck and hypomethylated for 25d_48h under the CHH context in the present study. BAK1 is an essential co-receptor of multiple receptor complexes, with well-characterized roles in the regulation of immunity and defense-related programmed cell death [70,71]. Interestingly, DMG *ClCG09G015110* encoding BAK1 was hypermethylated for 48h_ck under the CHH context in the present study, suggesting it functions in the early response to CGMMV infection in watermelon.

### 4.4. DMR-Associated Genes Enriched in Toll-Like Receptor Signaling During Cucumber Green Mottle Mosaic Virus Infection

Toll-like receptor signaling plays a central role in the immune response by recognizing pathogen-associated molecular patterns from bacteria and viruses [72]. Serine/threonine protein kinases (STKs) are receptor proteins that mediate signal transduction in plant defense. Serine/threonine protein kinases are mainly involved in the recognition and transduction of pathogen signals during the interaction of plants and microbes [73,74]. Four DMGs (*ClCG02G020720*, *ClCG07G002710*, *ClCG09G010710*, and *ClCG11G011240*) encoding STKs were identified in the Toll-like receptor signaling pathway in the present study. *ClCG07G002710* was hypomethylated for 48h_ck under the CG context and the other three DMGs were methylated under the CHH context. Receptor-like kinases/proteins (RLKs/RLPs) perceive and initiate PAMP-triggered immunity, which is the first layer of plant innate immunity [75]. In this study, six DMGs encoding RLKs/RLPs were identified. Except for *ClCG05G025640* (RLK3), which was hypomethylated for 48h_ck under the CG context, hypermethylated for 25d_ck under the CHH context, and hypomethylated for 25d_48h under CG and CHH contexts, the remaining DMGs were commonly methylated under the CHH context. These STKs and RLKs/RLPs play an important role in activating plant innate immunity.

### 4.5. DMR-Associated Genes Enriched in ABC Transporters During Cucumber Green Mottle Mosaic Virus Infection 

The ABC transporter is required to resist cell wall penetration and subsequent haustorium formation by the non-host barley powdery mildew pathogen *Blumeria graminis* f. sp. hordei (*Bgh*) and contributes to resistance to many other fungal and oomycete pathogens [76]. Six DMGs (*ClCG01G005380*, *ClCG02G007990*, *ClCG02G018950*, *ClCG09G018580*, *ClCG10G012900*, and *ClCG11G013200*) encoding ABC transporters were detected in the present study (Table S4). Except for *ClCG10G012900*, which was hypomethylated for 25d_48h under the CHG context, the remaining DMGs were methylated under the CHH context. These DMGs might contribute to resistance to CGMMV infection in watermelon. 

### 4.6. Relationship between DNA Methylation and Gene Expression 

Plant DNA methylation is interpreted by cells to maintain transposon silencing, promote chromatin structure, and ensure the normal expression of certain genes [20]. In *Arabidopsis*, correlations of methylation and expression differed between gene body methylation and TE methylation, gene body methylation is usually positively correlated and TE methylation is usually negatively correlated with expression [77]. In soybean, no obvious correlation between methylation changes and transcriptional variation of genes was detected based on transcriptional profiling [78]. In the present study, only 362 of the 2788 DMGs showed different expression levels in response to CGMMV infection (Table S5 and Figure 10). These differentially expressed DMGs were detected under the CG, CHG, and CHH contexts and the relationship between methylation changes and gene expression was unclear. In plants, only the methylation of gene bodies in the mCG context has been associated with higher gene expression, and more specifically, constitutive gene expression [26]. These methylated genes were characterized by the absence of mCG in and around the transcriptional start site and transcriptional termination site [79]. The methylation of gene regulatory elements also affects TF binding and gene expression. For example, in maize, higher mCHH levels upstream and downstream of genes have been associated with high gene expression [80]. Taken together, these findings suggest that much remains to be explored about DNA methylation and its impact on the regulation of gene expression.

### 4.7. RNA Interference and RdDM Pathways Associated with Watermelon Antiviral Processes

One of the first pieces of evidence for epigenomic regulation of plant immunity appeared with the description of the control of viral virulence through RNA silencing [81]. In tobacco, CHH hypomethylation was dominant at 16-day post-CMV infection, together with the loss of 24-nt siRNA distribution across the gene body [34]. In *Arabidopsis*, CMV infection with 2b deletion induced an enhanced population of 21-nt siRNAs and decreased the proportion of 24-nt siRNAs [82]. *Rice stripe virus* (*RSV*)-infected rice leaves revealed that siRNAs were derived almost equally from virion and the complementary RNA strands, and the length of siRNAs were mostly 20–22 nt long [83]. In our previous study, CGMMV infection induced the 21- and 22-nt siRNAs and reduced the level of 24-nt siRNAs in watermelon leaves [18]. Upon infection with the biotrophic fungal pathogen *Bgt*, AGO4a was significantly downregulated, a response similar to that in *Arabidopsis* plants elicited by the bacterial PAMP flg22 [37]. In this study, *AGO4* was also significantly downregulated during the process of CGMMV infection in watermelon (Table 3), which was accompanied by a substantial reduction in AGO4-sorted 24-nt siRNA levels as our previous study indicated [18]. 

DNA methylation mediated by 21-nt siRNAs may represent a different mechanism from the RdDM pathway, in which 24-nt siRNAs play a major role [84]. CHH methylation is usually mediated by RdDM pathway targeted in the short TEs and the edges of TEs [35]. In present study, CHH methylation occurs in most DMGs, suggesting the RdDM-directed CHH methylation plays important roles in watermelon response to the CGMMV. Genes encoding CMT, DRM2, RDR2, DCL3, AGO4, and AGO6 involved in the RdDM pathway were mostly downregulated after the CGMMV infection in the present study, which suggests depression of the RdDM pathway would initiate the immune response in watermelon. This observation was consistent with reduced DNA methylation, which promotes host defense responses against pathogen infection in wheat and *Arabidopsis* [35,83]. Thus, our results indicate reduced RdDM-directed DNA methylation of CHH and increased 21- and 22-nt siRNAs acting a synergetic effect on the antiviral process in watermelon. 

## 5. Conclusions

DNA methylation plays important roles in plant response to viral infection. In the present study, whole-genome bisulfite sequencing of watermelon leaves sampled at ck, 48 hpi, and 25 dpi were carried out. Methylation level at 48 hpi was lower than at ck and at 25 dpi. Most DMRs and DMGs were gathered under the CHH context during the process of CGMMV infection. DMGs involving in pathways of secondary biosynthesis and metabolism, plant–pathogen interactions, Toll-like receptor signaling, and ABC transporters were significantly enriched. Moreover, a range of DMGs encoding defense-related proteins were obtained. Correlation of DNA methylation and gene expression was analyzed by RNA-seq and no clear relationship was detected. Most genes involved in the RdDM pathway reduced their expression during the process of CGMMV infection. This study provides the DNA methylation variation in response to viral infection in watermelon for the first time. In addition, these results indicate the reduced RdDM-directed CHH methylation and increased 21- and 22-nt siRNAs act as a synergetic effect on antiviral defense in watermelon.

## Figures and Tables

**Table 1:** Summary of genome-wide methylation sequencing data

Terms	ck	48 hpi	25 dpi
Raw reads	91,070,116	80,314,902	73,989,862
Raw bases	13,660,517,400	11,098,479,300	12,047,235,300
Clean reads	83,839,958	71,659,074	67,011,742
Clean bases	12,402,189,963	9,905,599,786	10,592,716,908
Uniquely mapped reads	75,798,928	6,402,9094	60,394,949
Uniquely mapped rates (%)	90.41	89.35	90.13
Clean reads with unknown bases (Ns)	104,845	160,432	150,563
Quality score 20 (Q20) (%)	97.14	97.11	97.09
Quality score 30 (Q30) (%)	90.84	90.82	90.80
GC (%)	19.87	19.79	20.29
Coverage (%)	82.05	74.54	72.33
Strand depth	12.06	10.49	11.62
Depth >= 4 (%)	60.73	51.57	54.15
Conversion rate (%)	99.61	99.60	99.64

**Table 2:** DMRs identified in watermelon.

Comparison	DMRs Total	DMRs Hyper	DMRs Hypo	Intragenic DMRs	Intragenic Hyper	Intragenic Hypo	Intergenic DMRs	Intergenic Hyper	Intergenic Hypo
48h_ck_CG	205	23	182	39	11	28	166	12	154
48h_ck_CHG	205	29	176	41	11	30	164	18	146
48h_ck_CHH	16,073	10,631	5442	1293	860	433	14,790	9771	5009
48h_ck_Total	16,483	10,683	5800	1373	882	491	15,120	9801	5309
25d_ck_CG	61	19	42	11	3	8	50	16	34
25d_ck.CHG	96	63	33	31	21	10	65	42	23
25d_ck_CHH	24,193	23,004	1189	2112	2021	91	22,081	20,983	1098
25d_ck_Total	24,350	23,086	1264	2154	2045	109	22,196	21,041	1155
25d_48h_CG	181	28	153	28	6	22	153	22	131
25d_48h_CHG	215	12	203	36	3	33	179	9	170
25d_48h_CHH	30,127	3909	26,218	2502	292	2210	27,625	3617	24,008
25d_48h_Total	30,523	3949	26,574	2566	301	2265	27,957	3648	24,309

**Table 3:** Expression of genes involved in the RdDM pathway and RNA interference in watermelon.

Genes	Annotation	FPKM_ck	FPKM_48h	FPKM_25d	log_2_(48h_ck)	log_2_(25d_ck)	log_2_(25d_48h)
*ClCG08G009740*	DCL3	6.63	5.95	5.49	−0.16 (down)	−0.27 (down)	−0.11 (down)
*ClCG10G005250*	DCL3	4.08	5.28	2.96	0.37 (up)	−0.46 (down)	−0.84 (down)
*ClCG02G002110*	DCL3	1.49	1.83	0.00	0.30 (up)	Inf (down)	Inf (down)
*ClCG06G012100*	DCL4	2.37	2.63	1.78	0.15 (up)	−0.41 (down)	−0.56 (down)
*ClCG03G010530*	DCL2	5.64	5.38	21.89	−0.07 (down)	1.96 (up)	2.03 (up)
*ClCG02G024220*	AGO4	44.49	38.07	20.30	−0.23 (down)	−1.13 (down)	−0.91 (down)
*ClCG09G011170*	AGO6	1.40	0.70	0.00	−0.99 (down)	Inf (down)	Inf (down)
*ClCG00G004530*	AGO10	1.71	0.86	0.80	−1.00 (down)	−1.10 (down)	−0.10 (down)
*ClCG04G012520*	AGO1B	40.68	34.46	49.82	−0.24 (down)	0.29 (up)	0.53 (up)
*ClCG05G023990*	AGO	9.93	6.31	6.98	−0.65 (down)	−0.51 (down)	0.15 (up)
*ClCG05G004800*	AGO	40.68	34.46	49.82	−0.24 (down)	0.29 (up)	0.53 (up)
*ClCG01G014010*	AGO	5.19	6.18	6.52	0.25 (up)	0.33 (up)	0.08 (up)
*ClCG03G013970*	CMT	6.68	2.12	5.87	−1.65 (down)	−0.19 (down)	1.47 (up)
*ClCG10G001140*	CMT	15.47	17.87	14.80	0.21 (up)	−0.06 (down)	−0.27 (down)
*ClCG11G000400*	CMT	6.53	6.09	3.37	−0.10 (down)	−0.96 (down)	−0.85 (down)
*ClCG10G014050*	DRM2	20.84	19.90	14.12	−0.07 (down)	−0.56 (down)	−0.49 (down)
*ClCG06G004300*	DRM2	8.24	8.25	17.88	0.00	1.12 (up)	1.12 (up)
*ClCG01G006600*	RDR	0.06	0.41	0.00	2.76 (up)	Inf (down)	Inf (down)
*ClCG01G006450*	RDR	0.28	0.02	1.04	−4.04 (down)	1.86 (up)	5.90 (up)
*ClCG08G013320*	RDR6	7.14	4.69	1.40	−0.61 (down)	−2.35 (down)	−1.74 (down)
*ClCG07G005340*	RDR1a	0.86	1.12	1.18	0.38 (up)	0.46 (up)	0.07 (up)
*ClCG06G016860*	RDR2	3.61	2.93	1.83	−0.30 (down)	−0.98 (down)	−0.68 (down)

FPKM: Fragments per kilobase of exon per million reads.

## Supplementary Materials

The following are available online at https://www.mdpi.com/2073-4425/10/5/344/s1, Table S1: Differentially methylated regions (DMRs) identified in watermelon for 48h_ck, 25d_ck, and 25d_48h under CG, CHG, and CHH contexts, Table S2: Differentially methylated genes (DMGs) and their annotation identified in watermelon for 48h_ck, 25d_ck, and 25d_48h under CG, CHG, and CHH contexts, Table S3: KEGG pathway enrichment of DMGs for 48h_ck, 25d_ck and 25d_48h, Table S4: DMGs associated with CGMMV response in watermelon, Table S5: Expression of DMGs in response to CGMMV infection by RNA-Seq, Figure S1: The dynamics of genic DNA methylation for 15 selected CGMMV response-related DMGs using the IGV genome browser.
