# Supplementary material for: DNA Methylation Analysis of the Citrullus lanatus Response to Cucumber Green Mottle Mosaic Virus Infection by Whole-Genome Bisulfite Sequencing
Source: Genes (Basel). 2019 May 7;10(5):344. doi: 10.3390/genes10050344 (PMC6562589; doi:10.3390/genes10050344)

ClCG07G002280 (4-coumarate--CoA ligase-like 6)

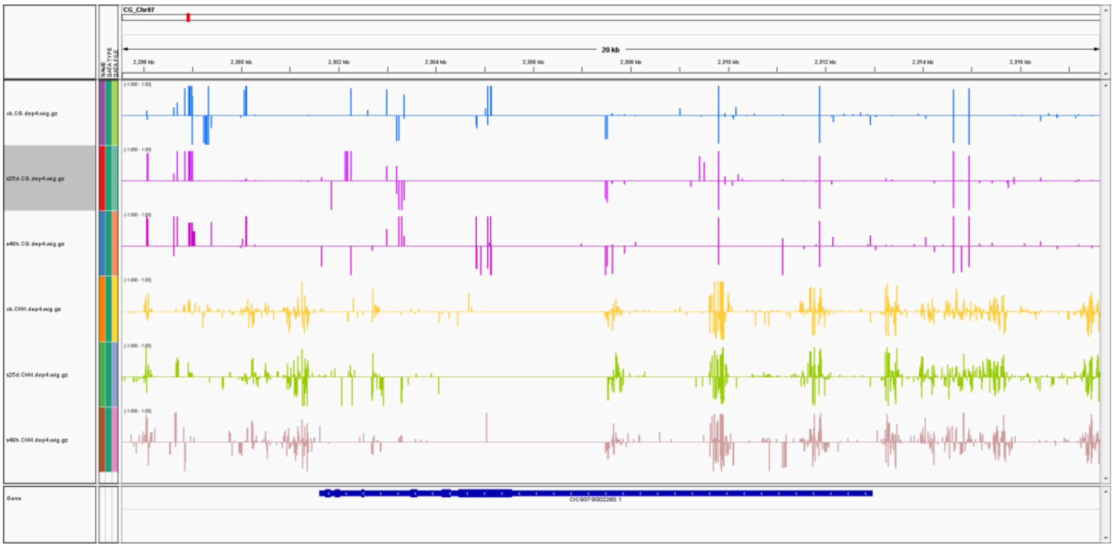

ClCG11G012560 (4-coumarate--CoA ligase 2-like)

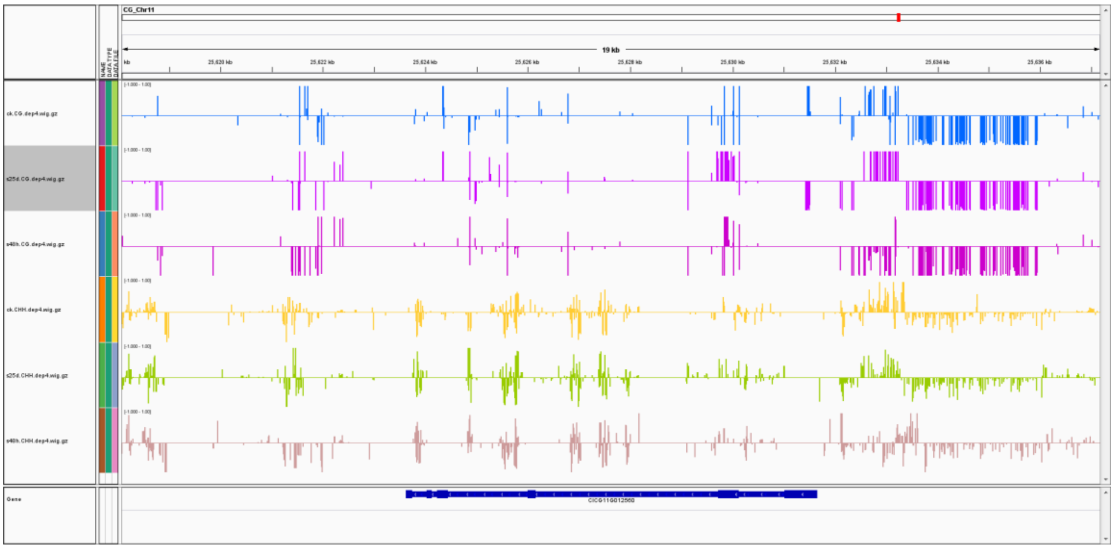

ClCG02G015680 (linoleate 9S-lipoxygenase 6-like)

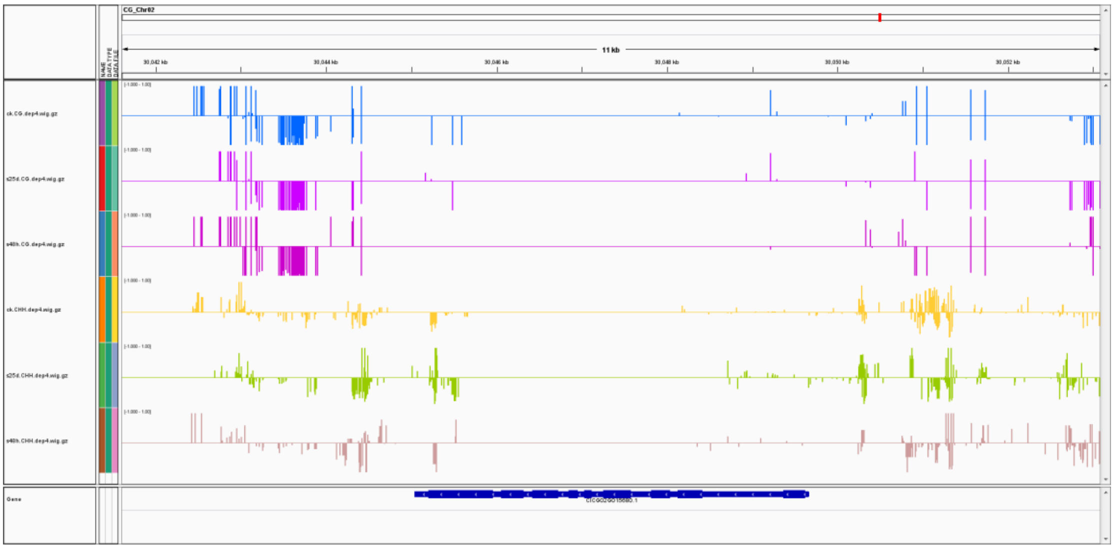

ClCG02G015640 (linoleate 9S-lipoxygenase 6-like)

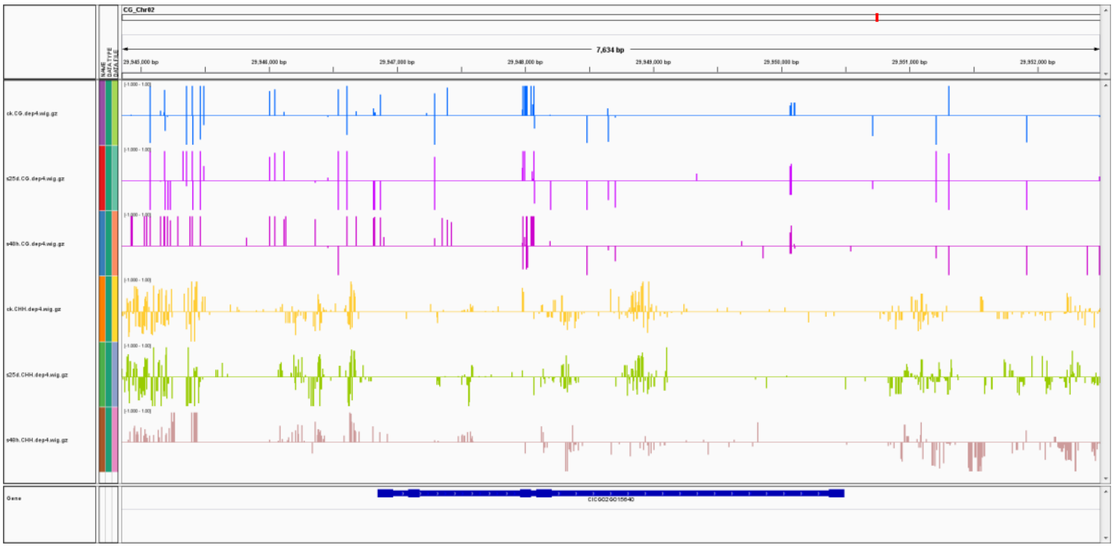

ClCG05G007400 (pectinesterase 2)

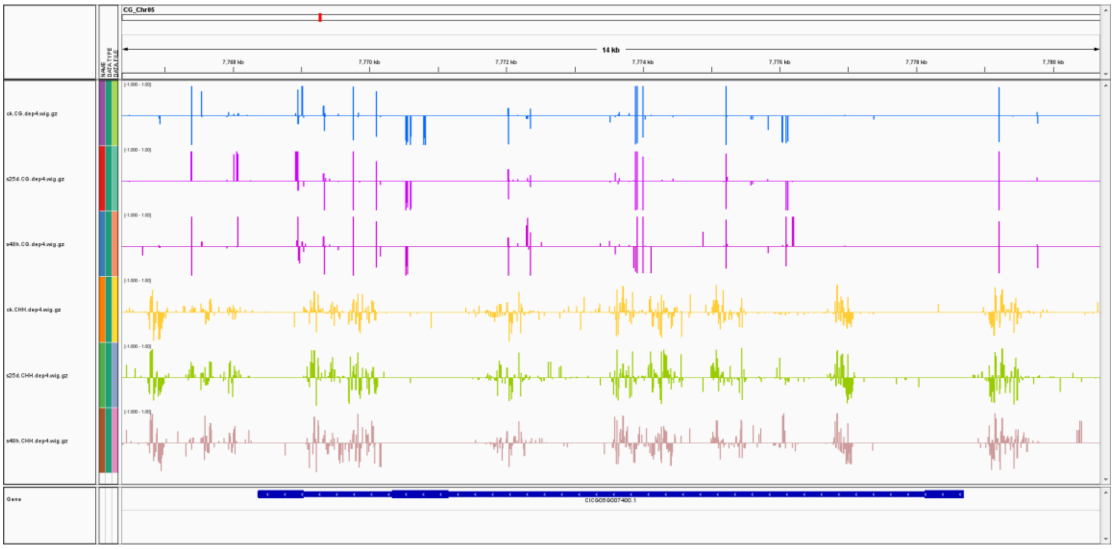

ClCG02G002690 (WRKY transcription factor 1 isoform X2)

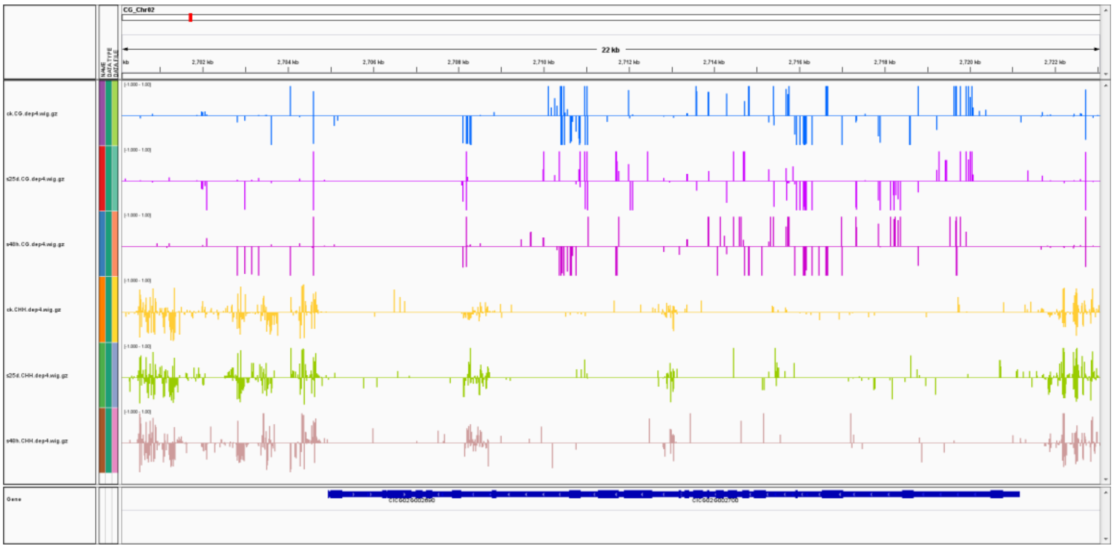

ClCG02G005880 (calmodulin-like protein 1)

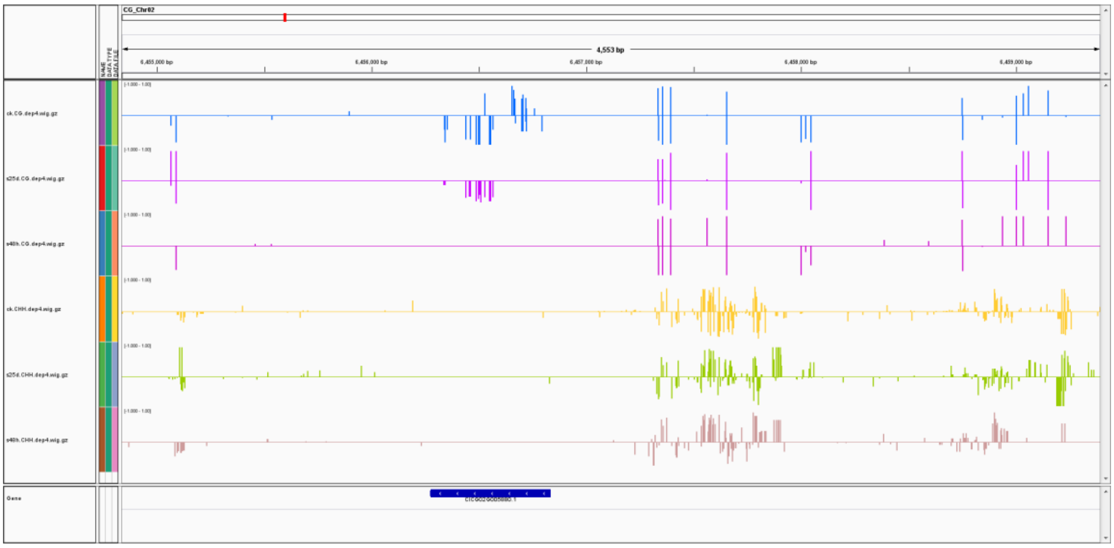

ClCG02G016580 (calmodulin-like protein 8)

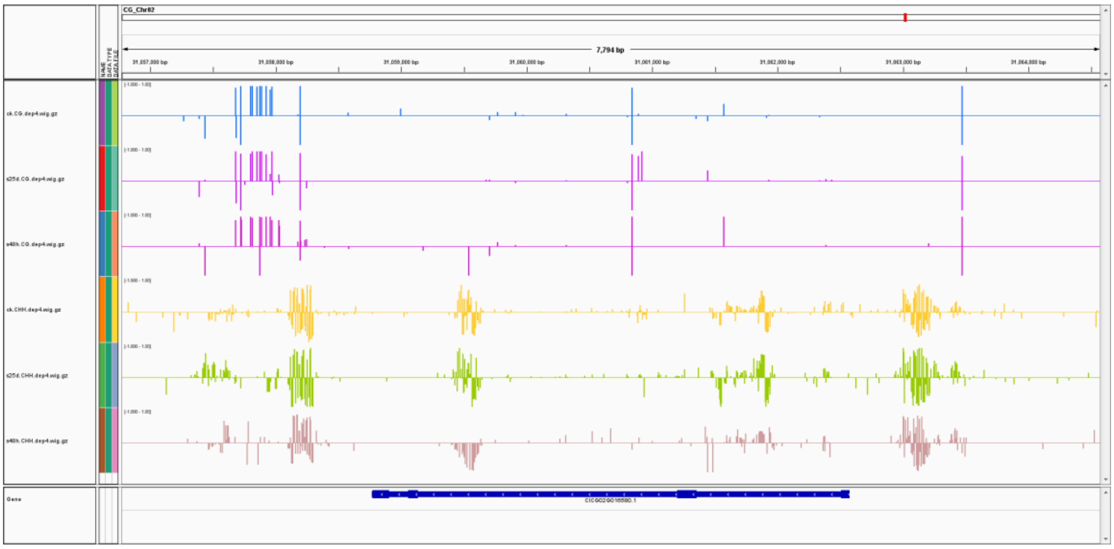

ClCG02G007230 (pathogenesis-related protein 1A-like)

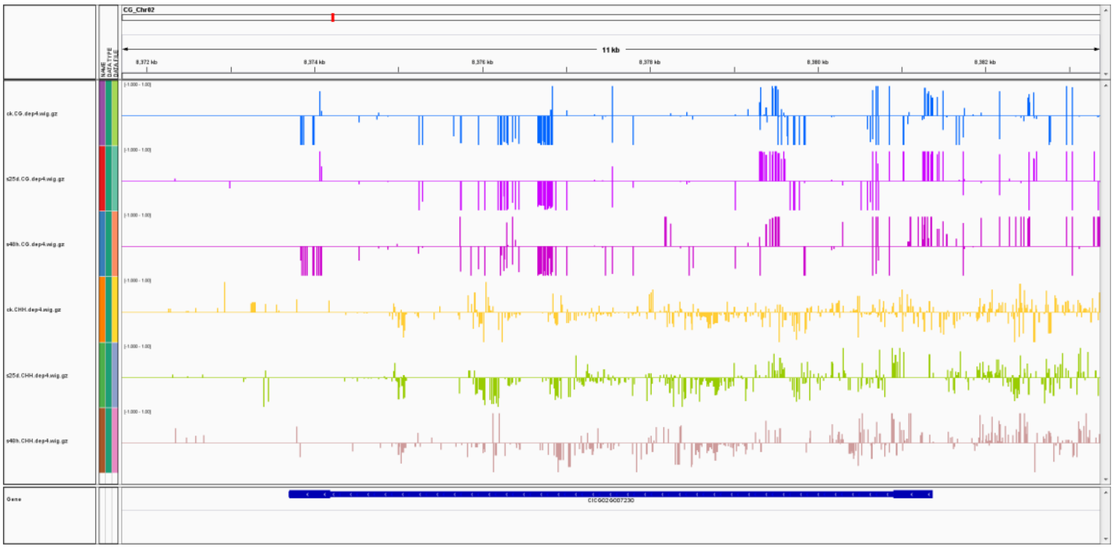

ClCG05G003210 (RPM1-interacting protein 4)

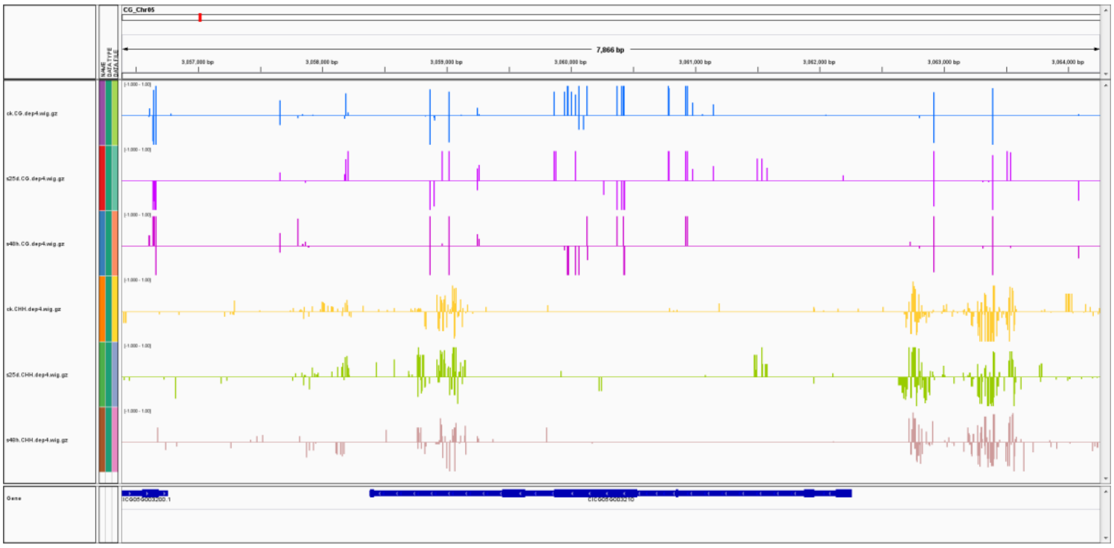

ClCG09G015110 (BRASSINOSTEROID INSENSITIVE 1-associated receptor kinase 1-like isoform X1)

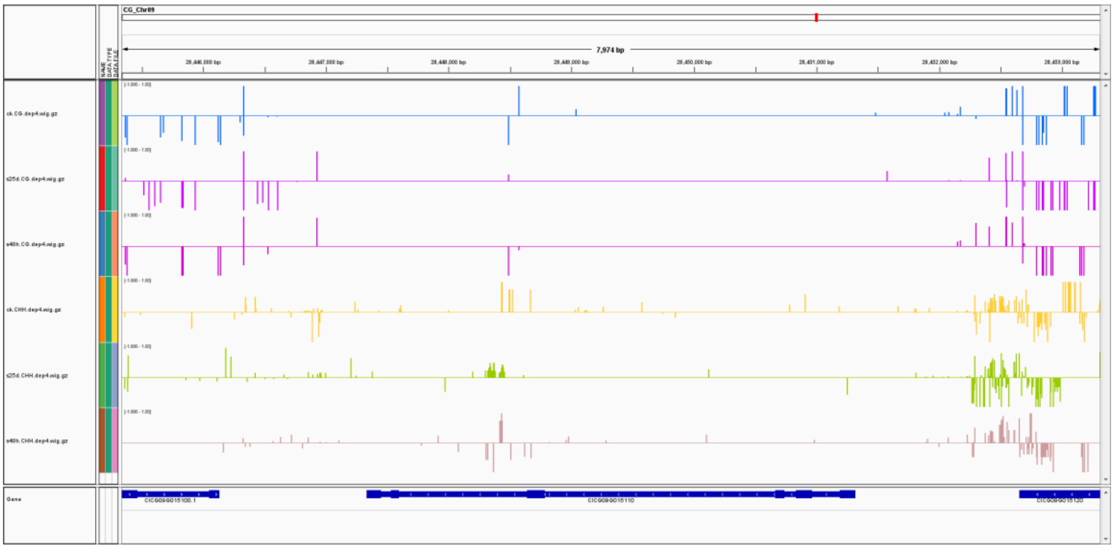

ClCG11G013200 (ABC transporter B family member 4)

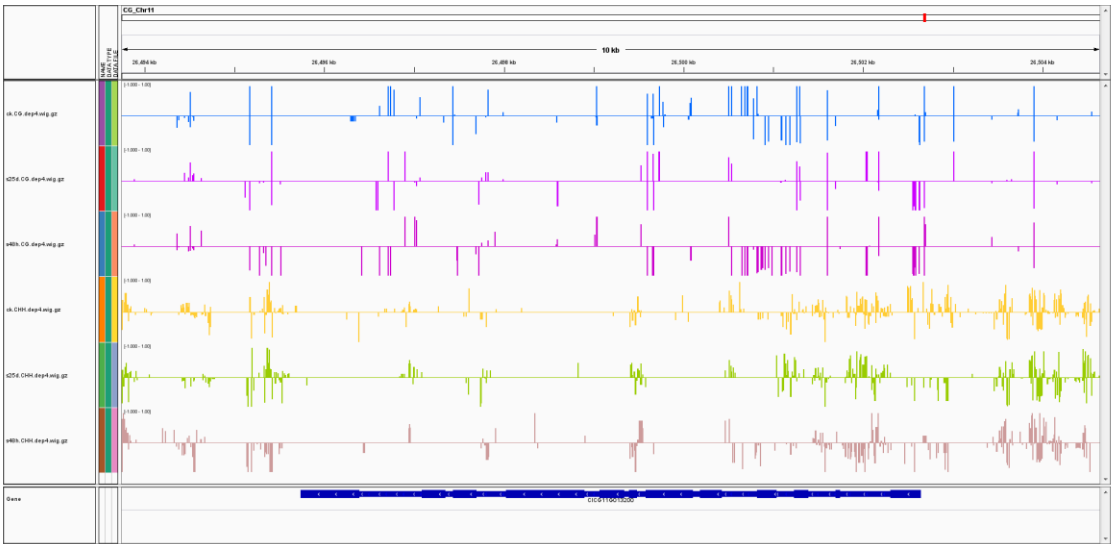

ClCG09G018580 (ABC transporter B family member 15-like)

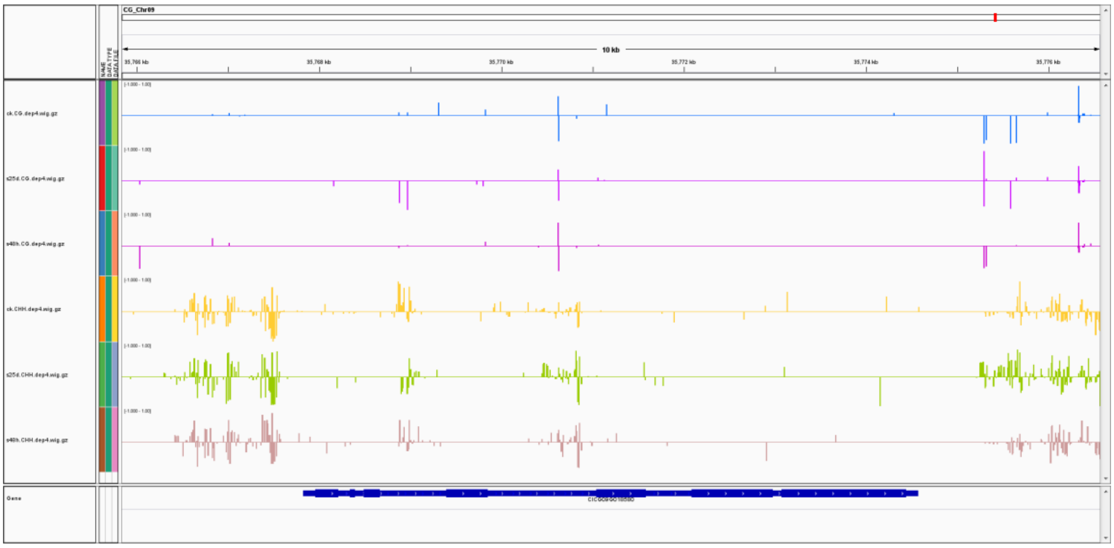

ClCG10G012900 (ABC transporter C family member 13 isoform X1)

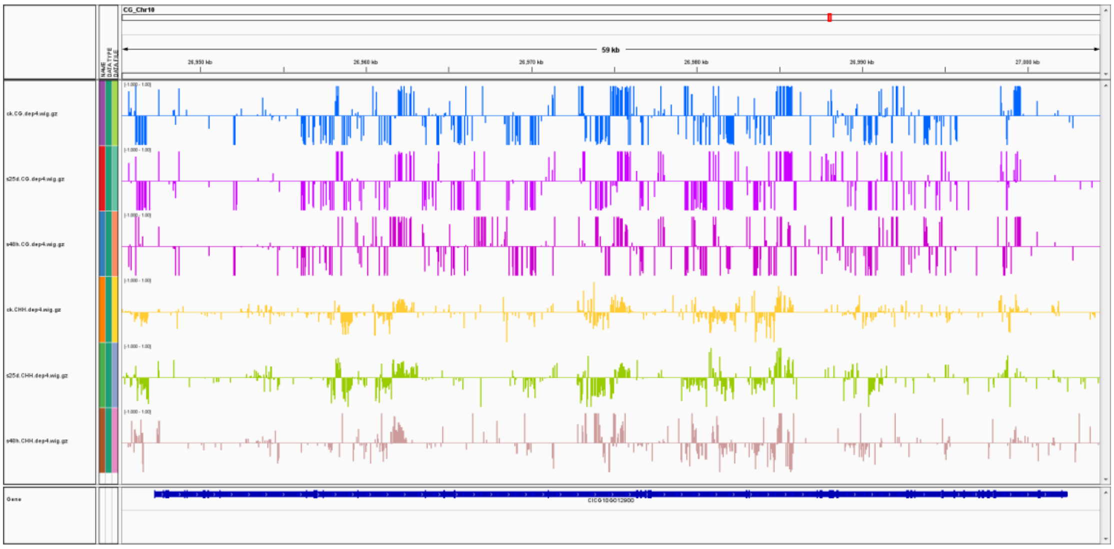

ClCG01G008480 (sucrose synthase 5)

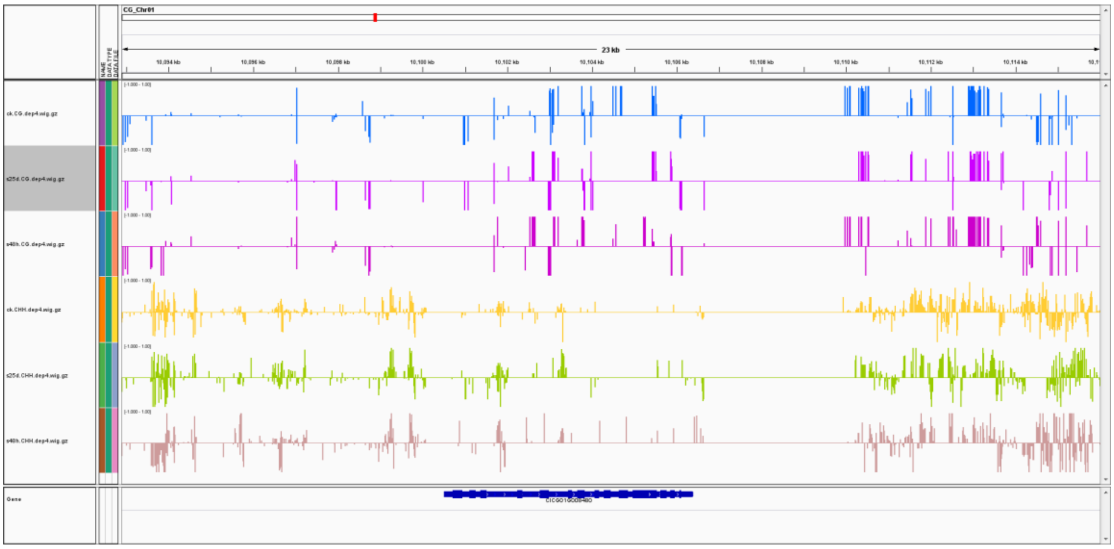

Supplement: Supplementary file 1 [file genes-10-00344-s001.zip › Supplementary files-final/Figure S1.pdf]
